# Qualität und Ethik in der Gesundheitsversorgung

**DOI:** 10.1007/s00103-022-03492-4

**Published:** 2022-02-07

**Authors:** Georg Marckmann, Jan Schildmann

**Affiliations:** 1 Institut für Ethik, Geschichte und Theorie der Medizin, Ludwig-Maximilians-Universität München, Lessingstr. 2, 80336 München, Deutschland; 2 Institut für Geschichte und Ethik der Medizin, Profilzentrum Gesundheitswissenschaften, Martin-Luther-Universität Halle-Wittenberg, Magdeburger Str. 8, 06112 Halle (Saale), Deutschland

**Keywords:** Ethik, Professionelles Handeln in der Medizin, Ressourcenallokation, Qualitätsindikatoren, Medizinische Ausbildung, Ethics, Medical professionalism, Resource allocation, Quality indicators, Medical education

## Abstract

Der Begriff „Qualität“ in der Gesundheitsversorgung wird häufig verwendet, aber unterschiedlich bestimmt. Einerseits beschreibt „Qualität“ die Beschaffenheit oder Eigenschaft von Dingen und ist in dieser Hinsicht deskriptiv. In den Bereichen Qualitätsmanagement und Qualitätssicherung steht aber die normative Dimension von „Qualität“ im Sinne der Bewertung von Strukturen, Prozessen oder Ergebnissen von Handlungen im Kontext der Gesundheitsversorgung im Mittelpunkt. Dabei bestehen verschiedene Anknüpfungspunkte zwischen ethischen Erwägungen im Gesundheitswesen und Qualität der Gesundheitsversorgung. Zunächst sind die Erbringung und Sicherung von hoher Qualität ein ethischer Imperativ, geboten durch die Prinzipien Wohltun und Nichtschaden. Für eine hohe ethische Qualität der Versorgung sind darüber hinaus aber auch die ethischen Prinzipien Achtung der Patient:innenautonomie und Gerechtigkeit zu berücksichtigen. Nicht zuletzt sind die Bestimmung und Begründung dessen, was „gute“ oder „hohe“ Qualität in der Gesundheitsversorgung meint, aus ethischer Sicht zu reflektieren. Der vorliegende Beitrag analysiert diese ethischen Dimensionen des Qualitätsmanagements und der Qualitätssicherung. Dazu wird zunächst erläutert, welche ethischen Anforderungen als Qualitätsmerkmale in der Patient:innenversorgung zu berücksichtigen sind. Anschließend werden ethisch relevante Herausforderungen bei der Bestimmung der Qualität im Gesundheitswesen anhand von Kriterien der Ergebnisqualität identifiziert und die Vermittlung professioneller Kompetenzen in der medizinischen Ausbildung als möglicher Beitrag zu Qualität und Qualitätssicherung im Gesundheitswesen erörtert. Den Abschluss bilden Überlegungen zur Bestimmung und Sicherung der Qualität unter den Bedingungen begrenzter Gesundheitsressourcen.

## Abstract (en)

The term “quality” in healthcare is frequently used but defined in different ways. On the one hand, quality describes the nature or characteristic of things and is descriptive in this respect. In quality management and quality assurance, however, the focus is on the normative dimension of quality, referring to the evaluation of structures, processes, or results of actions in the context of healthcare. There are several links between ethical considerations in healthcare and quality of healthcare. First, the provision and assurance of high quality is an ethical imperative, mandated by the principles of beneficence and nonmaleficence. However, for high ethical quality of care, the ethical principles of respect for patient autonomy and justice must also be considered. Last but not least, the determination and justification of what “good” or “high” quality in healthcare means must be reflected from an ethical perspective. This article analyses these ethical dimensions of quality management and quality assurance. To achieve this goal, it first explains which ethical requirements have to be considered as quality criteria in patient care. Subsequently, ethically relevant challenges in determining quality in healthcare are identified based on criteria of outcome quality, and the teaching of professional competencies in medical education is discussed as a possible contribution to quality and quality assurance in healthcare. The paper concludes with considerations on determining and assuring quality under conditions of limited healthcare resources.

## Einleitung

Der Begriff „Qualität“ gehört zu jenen, die häufig verwendet und gleichzeitig recht unterschiedlich bestimmt werden. Ausgehend von den aristotelischen Kategorien zum Wesen der Dinge bezeichnet „Qualität“ die Beschaffenheit oder auch Eigenschaft von Dingen und ist in dieser Hinsicht deskriptiv. In der Diskussion über Qualitätsmanagement und Qualitätssicherung steht freilich eine andere Lesart von Qualität im Vordergrund. Diese fokussiert auf die normative Dimension im Sinne der Bewertung von Strukturen, Prozessen oder Ergebnissen von Handlungen im Kontext der Gesundheitsversorgung. Die Verbindung zwischen der beschreibenden und bewertenden Dimension von Qualität ist insofern offenkundig, als sich Qualitätsmanagement und Qualitätssicherung auf deskriptive Merkmale von Strukturen, Prozessen und Ergebnissen stützen müssen, die mit einem vertretbaren Aufwand verlässlich erhoben werden können. Es muss also eine Auswahl von Merkmalen erfolgen, anhand derer die Qualität der Gesundheitsversorgung in einem normativen Sinne erfasst und dargestellt werden kann. Die Bestimmung und Bewertung von Qualität sind weiterhin häufig abhängig von der betrachtenden Person – und den jeweils zugrunde gelegten Bewertungsmaßstäben.

Angaben über die (normative) Qualität von Maßnahmen im Gesundheitswesen enthalten Urteile über eine „gute“, „erstrebenswerte“ beziehungsweise „richtige“ Art und Weise der gesundheitsbezogenen Versorgung von Menschen. Die objektive Analyse, Formulierung und Begründung solcher „Werturteile“ sind traditionell Aufgaben der Ethik. In dieser Hinsicht birgt die Diskussion über die Sicherung einer hohen Qualität im Gesundheitswesen verschiedene Anknüpfungspunkte zu ethischen Erwägungen. Zunächst sind die Erbringung und Sicherung von hoher Qualität ein ethischer Imperativ, geboten durch die Prinzipien Wohltun und Nichtschaden. Für eine hohe ethische Qualität der Versorgung sind darüber hinaus aber auch die Prinzipien Achtung der Patient:innenautonomie und Gerechtigkeit zu berücksichtigen. Schließlich sind die Bestimmung und Begründung dessen, was „gute“ oder „hohe“ Qualität in der Gesundheitsversorgung meint, aus ethischer Sicht zu reflektieren.

Zielsetzung des vorliegenden Beitrags ist die Reflexion dieser ethischen Dimensionen des Qualitätsmanagements und der Qualitätssicherung. Zunächst wird erläutert, welche ethischen Anforderungen als Qualitätsmerkmale in der Patient:innenversorgung zu berücksichtigen sind. Anschließend werden ethisch relevante Herausforderungen bei der Bestimmung der Qualität im Gesundheitswesen anhand von Kriterien der Ergebnisqualität identifiziert und die Vermittlung professioneller Kompetenzen in der medizinischen Ausbildung als möglicher Beitrag zu Qualität und Qualitätssicherung im Gesundheitswesen erörtert. Den Abschluss bilden Überlegungen zur Bestimmung der Qualität im Kontext begrenzter Ressourcen, die für die Gesundheitsversorgung zur Verfügung stehen.

## Erfüllung ethischer Anforderungen als Qualitätskriterien

An ärztliches Handeln wie auch die Praxis der Medizin sind nicht nur fachliche, sondern auch ethische Anforderungen gestellt. Diese sind historisch gewachsen und in weiten Bereichen erstaunlich stabil. Bereits in der Antike formulierte eine pythagoreische Ärzt:innengruppe einen Verhaltenskodex, der unter der Bezeichnung „hippokratischer Eid“ bis in die Neuzeit hineingewirkt hat. Mit der Schweigepflicht und den Geboten Nutzen und Nichtschaden enthält dieses historische Dokument moralische Verpflichtungen für Ärzt:innen, die auch heute noch verbindlich und beispielsweise in den ärztlichen Berufsordnungen sowie internationalen Kodizes festgeschrieben sind. Exemplarisch für einen solchen internationalen Kodex steht die 2002 veröffentlichte Physician Charter on Medical Professionalism [1]. Die vergleichsweise hohe Stabilität und Kontinuität der ethischen Anforderungen liegt wesentlich darin begründet, dass sich die Grundsituation von Not und Hilfe, die die Interaktion von Ärzt:innen und Patient:innen seit jeher prägt, im Verlauf der Zeit nicht grundsätzlich, sondern allenfalls graduell verändert hat. Patient:innen müssen in einer krankheitsbedingten Notsituation darauf vertrauen können, dass Ärzt:innen nicht nur fachlich kompetent sind, sondern darüber hinaus ihr Handeln an spezifischen moralischen Grundsätzen orientieren, die insbesondere sicherstellen, dass Ärzt:innen ihre eigenen Interessen zugunsten der Bedürfnisse der Patient:innen hintanstellen. Diese professionelle Selbstverpflichtung ist eine unverzichtbare Voraussetzung dafür, dass Patient:innen in existenziellen Notsituationen dem „System“ der ärztlichen Hilfe vertrauen können [2]. Die Selbstverpflichtung der Ärzt:innen ist Teil einer Vereinbarung zwischen Gesellschaft und ärztlicher Profession, die mit weitreichenden Privilegien und Freiheiten in Bezug auf fachliche, rechtliche und politische Ermessens- und Handlungsspielräume ausgestattet ist.

Aufgrund ihrer zentralen Bedeutung für die Interaktion zwischen Ärzt:innen und Patient:innen stellt die Erfüllung der ethischen Anforderungen auch ein wesentliches Qualitätsmerkmal der Patient:innenversorgung dar. Die Anforderungen sind in den international weithin anerkannten 4 medizinethischen Prinzipien des Wohltuns bzw. Nutzens, des Nichtschadens, der Achtung der Autonomie und der Gerechtigkeit kodifiziert [3]. Maßnahmen zur Qualitätssicherung sollten deshalb auch die Erfüllung der durch die Prinzipien vorgegebenen Anforderungen erfassen. Dies ist hinsichtlich der Prinzipien des *Nutzens* und *Nichtschadens* noch vergleichsweise einfach, da die etablierten Systeme der Qualitätssicherung in der Regel darauf abzielen, die positiven und negativen Effekte medizinischer Maßnahmen systematisch zu erfassen und zu steuern. Damit kann eine wesentliche Voraussetzung sichergestellt werden, dass Patient:innen die Behandlung mit dem bestmöglichen Nutzen-Schaden-Verhältnis angeboten wird. Allerdings müssen die positiven Effekte auf Grundlage patient:innenrelevanter Endpunkte erfasst werden, da nur dann gewährleistet ist, dass effektive Behandlungsmaßnahmen auch tatsächlich einen Nutzen für die Patient:innen bieten. Aus diesem Grund wird gefordert, dass die Bewertung neuer Substanzen weniger von Surrogatparametern, wie beispielsweise dem krankheitsfreien Überleben bei onkologischen Erkrankungen, abhängig sein sollte, sondern patient:innenrelevante Endpunkte wie das Gesamtüberleben und die Lebensqualität genutzt werden sollten, um zu erfassen, welchen Zusatznutzen Patient:innen von den Behandlungen haben [4, 5]. Dabei ist es auch wichtig, mittels Patient-reported Outcomes (PRO) die individuelle Wahrnehmung und Sichtweise der betroffenen Patient:innen, insbesondere auf ihre Lebensqualität, zu erfassen. Darüber hinaus müssen die Maßnahmen mit einem in klinischen Studien nachgewiesenen Nutzenpotenzial mit Blick auf die Besonderheiten des Einzelfalls so eingesetzt werden, dass sich das Nutzenpotenzial bei Patient:innen auch tatsächlich realisieren kann. Die Qualität der Indikationsstellung ist damit eine wesentliche Voraussetzung für die Realisierung der ethischen Prinzipien des Wohltuns und Nichtschadens.

Schwieriger ist das ethische Qualitätsmerkmal der *Achtung der Patient:innenautonomie* zu steuern. Bislang gibt es keine gut etablierten Instrumente, wie die Übereinstimmung der Behandlung mit den wohlinformierten Wünschen der betroffenen Patient:innen systematisch erfasst werden kann, zumal neben der Kongruenz von Behandlung und Patient:innenwille zudem die Qualität des zugrunde liegenden Entscheidungsprozesses mitberücksichtigt werden müsste. Die Bemühungen zur verbesserten Realisierung von Patient:innenautonomie fokussieren deshalb bislang vor allem auf die Förderung relevanter Prozessparameter selbstbestimmter Patient:innenentscheidungen. Beispielhaft erwähnt seien die partizipative Entscheidungsfindung (Shared Decision Making; [6]) oder Advance-Care-Planning-Programme zur Verbesserung der Vorausplanung von Behandlungsentscheidungen [7]. Beide Ansätze zielen darauf ab, Patient:innen stärker an der Entscheidungsfindung zu beteiligen und sie für selbstbestimmte Entscheidungen gemäß ihren individuellen Präferenzen zu befähigen. Dabei ist es nur konsequent, dass es zunehmende Bemühungen gibt, die Zielerreichung und damit Qualität von Shared Decision Making [8] und Advance Care Planning zu evaluieren [9].

Die ethische Anforderung von *Gerechtigkeit* im Zusammenhang mit Gesundheit und Krankheit als Qualitätskriterium gezielt zu erfassen und zu steuern, bietet ebenfalls Herausforderungen. Auf konzeptionell ethischer Ebene fehlt eine allgemein akzeptierte Theorie der Gerechtigkeit für den Gesundheitsbereich, aus der sich hinreichend konkret ableiten ließe, wie eine gesundheitliche Chancengleichheit und eine gerechte Patient:innenversorgung unter den Bedingungen begrenzter Ressourcen zu gestalten ist. Vergleichsweise gut lässt sich erfassen, ob es Einschränkungen im Zugang zu Gesundheitsleistungen gibt und welche Bevölkerungsgruppen bei den Gesundheitschancen benachteiligt sind.

Sozialepidemiologische Studien bieten dabei eine gute Möglichkeit, den Zusammenhang von sozialer Ungleichheit und Gesundheit zu erfassen [10]. Auf Grundlage dieser empirischen Erkenntnisse können dann konkrete Maßnahmen ergriffen werden, um die gerechtigkeitsethische Qualität der Gesundheitsversorgung zu erhöhen. Welche Anforderungen aus gerechtigkeitsethischer Sicht an die Patient:innenversorgung unter den Bedingungen der Ressourcenknappheit gestellt sind, wird unten im Abschnitt zur Qualitätssicherung unter Bedingungen der Ressourcenknappheit noch einmal aufgegriffen.

Eine Möglichkeit, die Qualität von Indikationsstellung und Versorgung mit dem Fokus auf ethische Anforderungen zu reflektieren, stellen ethische Fallberatungen dar. So steht im Rahmen der prinzipienorientierten Ethikfallberatung das mögliche Nutzen- und Schadenspotenzial laufender beziehungsweise avisierter Maßnahmen im Zentrum der Analyse aus der Fürsorgeperspektive [11]. Voraussetzung für einen solchen Beitrag von Ethikfallberatung zur Qualität in der Gesundheitsversorgung ist die qualifizierte Durchführung entsprechender Beratungsangebote, deren Qualität zunehmend Gegenstand von Evaluationsstudien ist [12, 13].

## Kriterien zur Bestimmung der Ergebnisqualität: Ethische Herausforderungen

Die Bestimmung von messbaren erwünschten Outcomes ist zentraler Bestandteil der Bemühungen um Qualität und Qualitätssicherung in der Gesundheitsversorgung. So stellt die Umsetzung von und Beteiligung an einrichtungsübergreifenden Maßnahmen, die die Verbesserung der Ergebnisqualität zum Ziel haben, nach § 135a Fünftes Buch Sozialgesetzbuch (SGB V) eine Priorität dar. Aus ethischer Perspektive ist der hohe Stellenwert dieser Art von Qualität nicht zuletzt unter Verweis auf die bereits erwähnten ethischen Prinzipien wie Wohltun und Nichtschaden gut zu begründen. Maßnahmen der Gesundheitsversorgung, die *im Ergebnis* nicht beiden Prinzipien angemessen Rechnung tragen, scheinen weder qualitativ noch ethisch angemessen.

Ethisch relevante Herausforderungen für die Definition und Begründung von Kriterien zur Bestimmung der Qualität in der Gesundheitsversorgung ergeben sich insbesondere dann, wenn der Einsatz einer medizinischen Maßnahme und diesbezügliche Outcomes nur schwerlich in aggregierter Form bewertet werden können. Exemplarisch hierfür steht die Verwendung von Qualitätsindikatoren zur Bewertung der Versorgung von fortgeschritten an Krebs erkrankten Patient:innen. So wurden bereits 2003 auf der Grundlage von Arbeiten von Earle et al. [14] Qualitätsindikatoren für die Versorgung in der letzten Lebensphase definiert, die auf der Grundlage von Daten aus der Routinedokumentation gewonnen werden können. Ein Beispiel für einen solchen Qualitätsindikator ist die Durchführung intensivmedizinischer Behandlungsmaßnahmen in den letzten 30 Lebenstagen. Während es durchaus Anhaltspunkte dafür gibt, die Durchführung intensivmedizinischer Maßnahmen in der letzten Lebensphase auch unter dem Gesichtspunkt der Qualität der Gesundheitsversorgung kritisch zu hinterfragen, ergibt sich die ethische Bewertung der Praxis am Lebensende nicht allein aus der Tatsache, ob eine intensivmedizinische Behandlung in den letzten Lebenstagen durchgeführt wurde oder nicht. Vielmehr wäre es ethisch problematisch, wenn die Anwendung des oben genannten Qualitätsindikators dazu führen würde, dass Patient:innen mit einer fortgeschrittenen Tumorerkrankung und einem Risiko, im Verlauf des nächsten Monats zu versterben, per se nicht mehr intensivmedizinisch behandelt würden. Ethisch zu rechtfertigen wäre ein Verzicht solcher Maßnahmen nur dann, wenn diese Maßnahmen nicht durch den Willen der betreffenden Patient:innen gedeckt wären beziehungsweise wenn entsprechend der professionellen ärztlichen Bewertung des möglichen Nutzens und Schadens der Maßnahme keine Indikation für eine intensivmedizinische Behandlung gestellt würde. Ähnlich verhält es sich, wenn in der Diskussion über Qualität der Krebsversorgung die Übereinstimmung von Tumorkonferenzempfehlung und tatsächlich durchgeführter tumorspezifischer Behandlung als Maßstab herangezogen wird. Die interdisziplinäre Falldiskussion über eine tumorspezifische Therapie stellt sowohl strukturell als auch prozessual ein wichtiges Qualitätsmerkmal dar, welches das Patient:innenwohl unterstützen kann. Allerdings kann es aus ethischer Perspektive gute Gründe für die Abweichung der tatsächlichen Behandlung von der empfohlenen Behandlung geben. Dies gilt beispielsweise dann, wenn Ärzt:innen und Patient:innen im Rahmen der individuellen Entscheidungsfindung zum Ergebnis gelangen, dass eine andere Handlungsoption den Präferenzen der Patient:innen eher entspricht.

Den beiden vorstehenden Beispielen gemeinsam ist, dass die Definition empirisch robuster Kriterien zur Bestimmung von Qualität normative Setzungen enthalten kann, die sich im Einzelfall unter ethischen Gesichtspunkten als problematisch darstellen. Für beide Qualitätskriterien relevant ist dabei, dass Wertungen aus der Perspektive der Patient:innen nicht abgebildet werden. Während der Verzicht auf eine intensivmedizinische Behandlung in einem Teil der Fälle unter Verweis auf eine fehlende Indikation ethisch begründet werden könnte, kann die Durchführung einer Therapie sich nie allein auf medizinische Expertise stützen, sondern muss immer durch den Willen von Patient:innen gedeckt sein. Eine ethisch reflektierte Adaption der genannten Kriterien könnte dahin gehend erfolgen, dass das Handlungsergebnis in Verbindung mit einer Information zum (mutmaßlichen oder vorausverfügten) Patient:innenwillen erfasst wird. Die Durchführung intensivmedizinischer Maßnahmen bei fortgeschritten erkrankten Menschen ohne Anhalt für einen entsprechenden Patient:innenwillen wäre ein Indikator für eine ethisch (und rechtlich) problematische Gesundheitsversorgung. Diese Überlegungen verdeutlichen, dass die Erfassung der Qualität der Patient:innenversorgung nicht auf einzelne quantifizierbare und leicht messbare Parameter des Behandlungserfolgs reduziert werden kann. Vielmehr sind komplexere Qualitätskonstrukte erforderlich, um angemessen zu erfassen, inwieweit sich die Versorgung am Ideal einer patient:innenzentrierten Medizin orientiert.

## Professionelle Kompetenzen als Voraussetzung für die Qualität der Patient:innenversorgung

Die Qualität der Aufklärung von Patient:innen über eine medizinische Behandlung oder des Umgangs mit moralischen Unsicherheiten oder Konflikten sind Beispiele für wichtige Kriterien der Prozessqualität der Gesundheitsversorgung. Gleichzeitig stellt die Erfassung der Qualität solcher komplexen Praktiken methodisch eine Herausforderung dar. Es stellt sich weiterhin die Frage, wie gewährleistet werden kann, dass die Akteur:innen im Gesundheitswesen diese Aspekte von Qualität in ihrer Tätigkeit berücksichtigen können.

Ein diesbezüglich relevantes und zunehmend auch in Deutschland diskutiertes Konzept ist die Aus- und Fortbildung „professioneller Kompetenzen“. Ausgehend von der Diskussion über „medical professionalism“ [15] im angelsächsischen Sprachraum können unter professionellem Verhalten insbesondere die Kompetenzen zusammengefasst werden, die ergänzend zu medizinisch fachlichem Wissen und Fertigkeiten die ethischen, rechtlichen und kommunikativ relevanten Aspekte der Gesundheitsversorgung betreffen. Während die Inhalte professionellen Handelns zeitlich und kulturell kontingent sind, bilden die Vermittlung und der Erwerb entsprechender Fertigkeiten und Kenntnisse die Voraussetzung dafür, dass auf der Ebene der Akteur:innen eine professionelle und in diesem Sinne auch qualitativ hochwertige Gesundheitsversorgung verankert werden kann. Ergänzend zur Debatte über professionelles Verhalten hat in den letzten Jahren eine Diskussion über Möglichkeiten der Ausbildung einer „professionellen Identität“ Beachtung gefunden. Im Mittelpunkt stehen Merkmale, Werte und Normen des ärztlichen Berufs, die im Rahmen der individuellen Entwicklung beziehungsweise kollektiven Sozialisierung erworben werden und als relevant für das professionelle Handeln als Ärzt:in [16] erachtet werden.

Die Bedeutung professionellen Handelns für eine qualitativ hochwertige Gesundheitsversorgung und diesbezügliche relevante Kompetenzen und Werte wurden in der bereits zitierten ärztlichen Charta zur Berufsethik zusammengefasst [1]. Die Charta umfasst neben Grundprinzipien wie dem Vorrang des Patient:innenwohls, der Patient:innenautonomie und der sozialen Gerechtigkeit auch berufliche Verpflichtungen, die für die Qualität der Gesundheitsversorgung von Bedeutung sind. Exemplarisch stehen hierfür die Verpflichtung zur Verbesserung der Versorgungsqualität und des Zugangs zur Versorgung sowie der angemessene Umgang mit Interessenkonflikten.

Die Verknüpfung zwischen Qualität in der Gesundheitsversorgung und professionellem Handeln spiegelt sich auch in der im April 2021 veröffentlichten Version 2.0 des Nationalen Kompetenzbasierten Lernzielkatalogs Medizin (NKLM) wider [17]. Der Katalog, der im Anschluss an die derzeit vom Medizinischen Fakultätentag koordinierte Weiterentwicklung und mit Inkrafttreten der neuen ärztlichen Approbationsordnung die Grundlage für die Lehre an den medizinischen Fakultäten in Deutschland bilden soll, enthält zum einen Lernziele, die sich mit Kenntnissen und Fertigkeiten zum Qualitätsmanagement befassen (Tab. 1).

**Table 1:** 

VIII.5-05	Die Absolventin und der Absolvent kennen Modelle und Methoden des Qualitätsmanagements und wenden diese an
VIII.5-05.1	Sie haben Kenntnis über Maßnahmen zur Qualitätssicherung in der Patientenversorgung und deren Anwendungsbereiche

Zum anderen umfasst der NKLM Lernziele zum professionellen Handeln, die von Relevanz für die Qualität der Gesundheitsversorgung sind. Exemplarisch stehen hierfür Lernziele aus dem Kapitel „Professionelles Handeln, Ethik, Geschichte und Recht der Medizin“ (Tab. 2).

**Table 2:** 

VIII.6-01.3.1	Den ärztlichen Verantwortungsbereich definieren und das eigene Handeln daran ausrichten
VIII.6-01.3.2	In ihrer professionellen Rolle angemessene Beziehungen zu Patientinnen und Patienten, Angehörigen, Kolleginnen und Kollegen, Pflegenden und anderen Berufsgruppen gestalten

Die Auswahl geeigneter Methoden zur Vermittlung und Prüfung entsprechender Kompetenzen unter den gegebenen Rahmenbedingungen für die medizinische Ausbildung stellt eine wichtige Aufgabe dar. Hinzu kommt, dass der postulierte Zusammenhang zwischen professionellem Verhalten und einer qualitativ hochwertigen Gesundheitsversorgung evaluiert werden muss.

## Qualität der Versorgung unter Bedingungen begrenzter Ressourcen

Die Realisierung einer qualitativ hochwertigen Patient:innenversorgung erfordert materielle und personelle Ressourcen, wobei der zusätzliche Ressourcenaufwand mit zunehmendem Qualitätsniveau in vielen Bereichen überproportional ansteigen dürfte. Unter den aktuellen Rahmenbedingungen ist davon auszugehen, dass die Gesundheitsversorgung in Deutschland auf absehbare Zeit unter den Bedingungen begrenzt verfügbarer Ressourcen zu organisieren ist. Medizinische Innovationen steigern in einer alternden Bevölkerung die Gesundheitsausgaben, während die Einnahmen in der umlagefinanzierten gesetzlichen Krankenversicherung durch den steigenden Altenquotienten sinken. Es ist deshalb davon auszugehen, dass nicht in allen Bereichen die medizinisch bestmögliche Qualität der Patient:innenversorgung finanziert werden kann. Damit stellt sich die – auch ethisch relevante – Frage, welches Qualitätsniveau in den verschiedenen Versorgungsbereichen erreicht werden kann beziehungsweise soll. Zudem ist aus ethischer Sicht zu gewährleisten, dass das jeweils erreichte Qualitätsniveau über verschiedene Patient:innengruppen hinweg einigermaßen gleich verteilt ist. Eine explizite Priorisierung des Ressourceneinsatzes nach klar definierten, ethisch begründeten Kriterien kann dazu beitragen, dass mit den begrenzt verfügbaren Ressourcen die vorrangigen Versorgungsbedarfe gedeckt werden und eine gleichmäßige Versorgung der Bevölkerung gewährleistet ist [18]. Insbesondere ist zu vermeiden, dass eine qualitativ hochwertige Versorgung nur in finanziell lukrativen Versorgungsbereichen angeboten wird. Entsprechende Anreize im Vergütungssystem sind zu korrigieren.

Zugleich sollte unter den gegebenen Rahmenbedingungen der sorgsame Umgang mit den begrenzt verfügbaren Ressourcen zu einem Qualitätsmerkmal der Versorgung werden. Dies beinhaltet insbesondere die Vermeidung von Über- und Fehlversorgung, wie sie im Gesundheitswesen an vielen Stellen anzutreffen ist [19]. Es ist deshalb zu begrüßen, dass sich medizinische Fachgesellschaften mit Initiativen wie „Choosing Wisely“ [20] oder „Gemeinsam klug entscheiden“ [21] zunehmend des Problems der Überversorgung annehmen und Empfehlungen erarbeiten, welche medizinischen Maßnahmen in welchen Situationen wegen eines fehlenden Zusatznutzens für die Patient:innen nicht eingesetzt werden sollten [22]. Der sorgsame Umgang mit begrenzt verfügbaren Ressourcen lässt sich als wesentliches Qualitätsmerkmal unter Knappheitsbedingungen ebenfalls nicht leicht valide erfassen, da die richtige Indikationsstellung nur mit großem Aufwand im Nachhinein geprüft werden kann. Auch hier sind deshalb die professionellen Kompetenzen des Gesundheitspersonals besonders gefordert: Wie können Ärzt:innen in einer ethisch vertretbaren Art und Weise Ressourcenerwägungen in ihren Entscheidungen berücksichtigen [23]? Zudem sollte die Berücksichtigung ethischer Vorgaben insbesondere unter dem zunehmenden ökonomischen Druck zu einem integralen Bestandteil des Managements von Gesundheitseinrichtungen werden – im Sinne eines Wertemanagements [24]. Das werteorientierte Verhalten der Mitarbeitenden kann als „innere Qualität“ der „äußeren Qualität“, d. h. der Qualität der Behandlungsergebnisse, gegenübergestellt werden [25]. Zu den relevanten Werten gehören u. a. Patient:innenorientierung, Mitarbeiter:innenorientierung, Führungsqualität oder der sorgsame Umgang mit Ressourcen. Maßnahmen zur Qualitätssicherung sollten entsprechend auch die „innere Qualität“ von Gesundheitseinrichtungen erfassen und damit die Voraussetzungen sichern, dass auch unter schwierigen finanziellen Rahmenbedingungen eine im umfassenderen Sinne hohe Qualität der Patient:innenversorgung realisiert werden kann. Als Instrumente des Qualitätsmanagements kommen hier vor allem (wiederholte) Befragungen von Mitarbeitenden in Betracht, da sich die personenbezogenen Determinanten der „inneren Qualität“ nur über die Einschätzung von Personen erfassen lassen.

Die Maßnahmen zur Qualitätssicherung selbst sind ebenfalls darauf zu prüfen, ob sie einen gerechtfertigten Mitteleinsatz darstellen, da sie mit der Patient:innenversorgung selbst um prinzipiell begrenzte Ressourcen konkurrieren. Insofern sind Maßnahmen zur Qualitätssicherung immer auch auf das Verhältnis von Ressourcenaufwand und Nutzen für die Patient:innenversorgung zu prüfen. Weniger aufwendige Maßnahmen sind zu bevorzugen, auch mit Blick auf die in der Regel erforderliche zusätzliche Dokumentation und Kontrolle des Versorgungsgeschehens. Hier sei noch einmal auf die professionellen Pflichten des Gesundheitspersonals verwiesen („medical professionalism“): Je besser das Personal seinen Berufspflichten nachkommt, je besser die erforderlichen professionellen Kompetenzen ausgebildet sind, desto weniger werden Qualitätssicherungsmaßnahmen erforderlich, die mit einem hohen bürokratischen Aufwand das Versorgungsgeschehen dokumentieren und kontrollieren – was insbesondere unter den aktuellen Bedingungen der Personalknappheit mit relevanten Opportunitätskosten und ggf. auch Einbußen bei der Qualität der Patient:innenversorgung verbunden sein kann.

## Fazit

Bemühungen zur Sicherung und Förderung der Qualität der Patient:innenversorgung weisen verschiedene ethisch relevante Dimensionen auf. Zunächst stellt die Erfüllung ethischer Anforderungen, wie sie in den weithin anerkannten medizinethischen Prinzipien des Nutzens, Nichtschadens, Achtung der Autonomie und der Gerechtigkeit formuliert sind, wesentliche Qualitätsmerkmale der Patient:innenversorgung dar. Die Qualität der Patient:innenversorgung darf deshalb nicht auf quantifizierbare und leicht zu erfassende Parameter reduziert werden, sondern erfordert mehrdimensionale Konstrukte, die sich umfassender am Leitbild einer patient:innenzentrierten Medizin orientieren. Die ethischen Qualitätsdimensionen lassen sich dabei in der Regel schwieriger erfassen und gezielt steuern. Prozessuale Maßnahmen zur Förderung der ethischen Versorgungsqualität wie Shared Decision Making, Advance Care Planning oder Ethikberatung erscheinen hierfür ebenso erforderlich wie eine Stärkung professioneller Kompetenzen des Gesundheitspersonals im Rahmen der Aus‑, Fort- und Weiterbildung. Insbesondere unter den Bedingungen begrenzt verfügbarer Ressourcen kann eine im umfassenderen Sinne gute Versorgungsqualität nur erreicht werden, wenn das Gesundheitspersonal hierfür professionelle Verantwortung übernimmt. Zudem müssen die Rahmenbedingungen der Patient:innenversorgung auf der Makro- und Mesoebene so gestaltet werden, dass die „weichen“, ethisch aber höchst relevanten Faktoren einer qualitativ hochwertigen Patient:innenversorgung insbesondere unter dem aktuellen ökonomischen Druck im Gesundheitswesen nicht vernachlässigt werden. Beispielhaft erwähnt sei hier die Sicherung der Werteorientierung in Gesundheitseinrichtungen. Weiterer Forschungsbedarf besteht hinsichtlich der Frage, welche Maßnahmen geeignet und nachgewiesen effektiv sind, diese unverzichtbaren systemischen Struktur- und Prozessparameter einer guten Patient:innenversorgung zu fördern.
